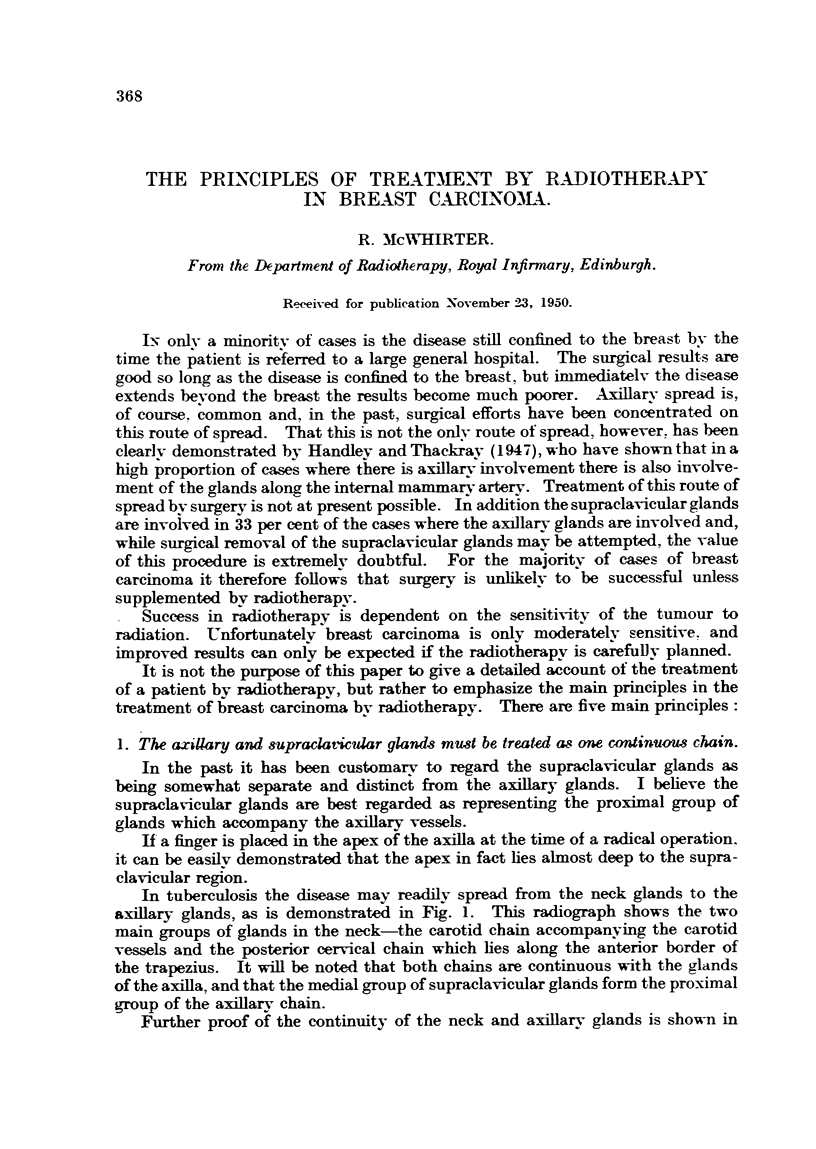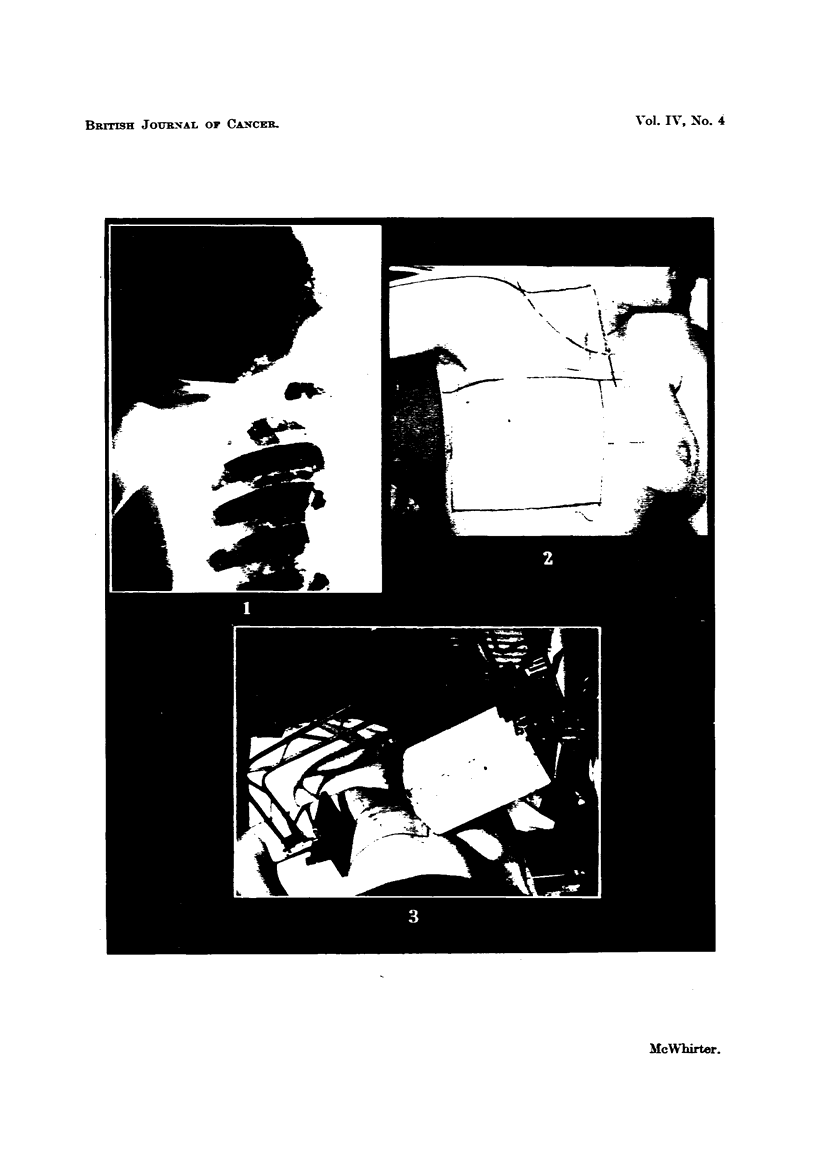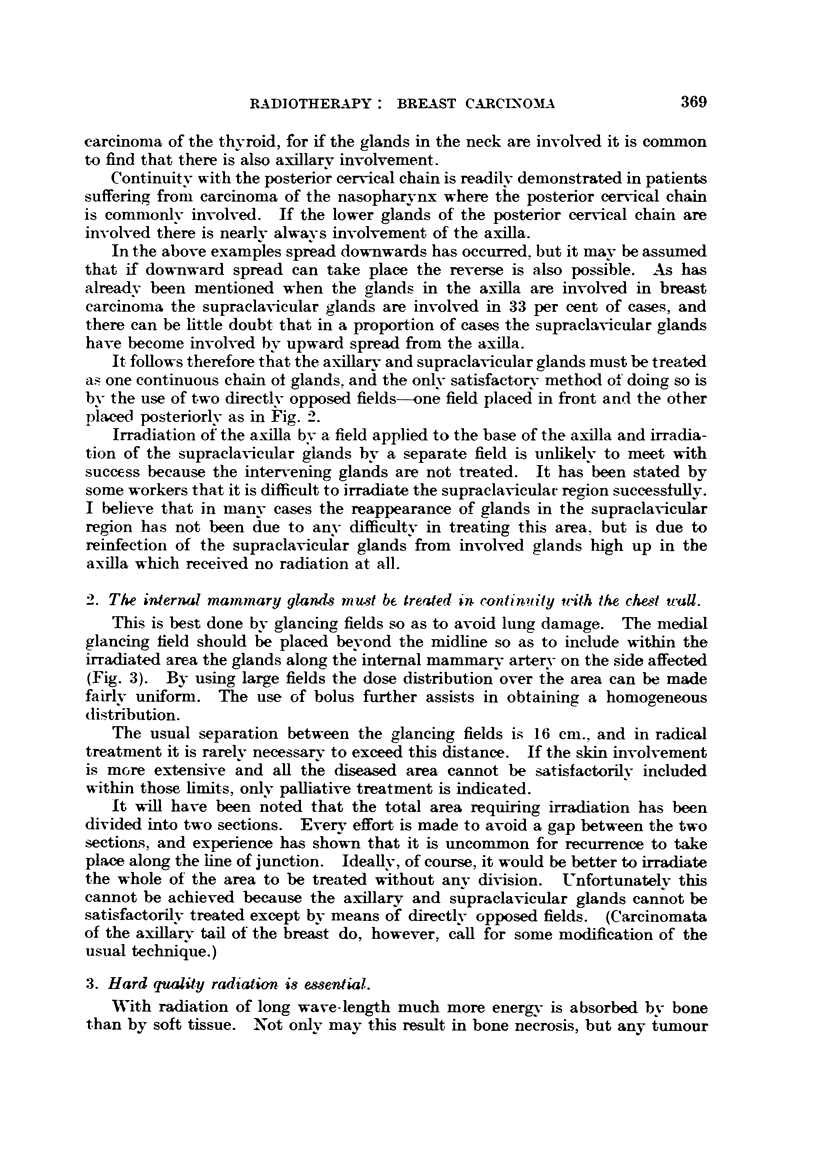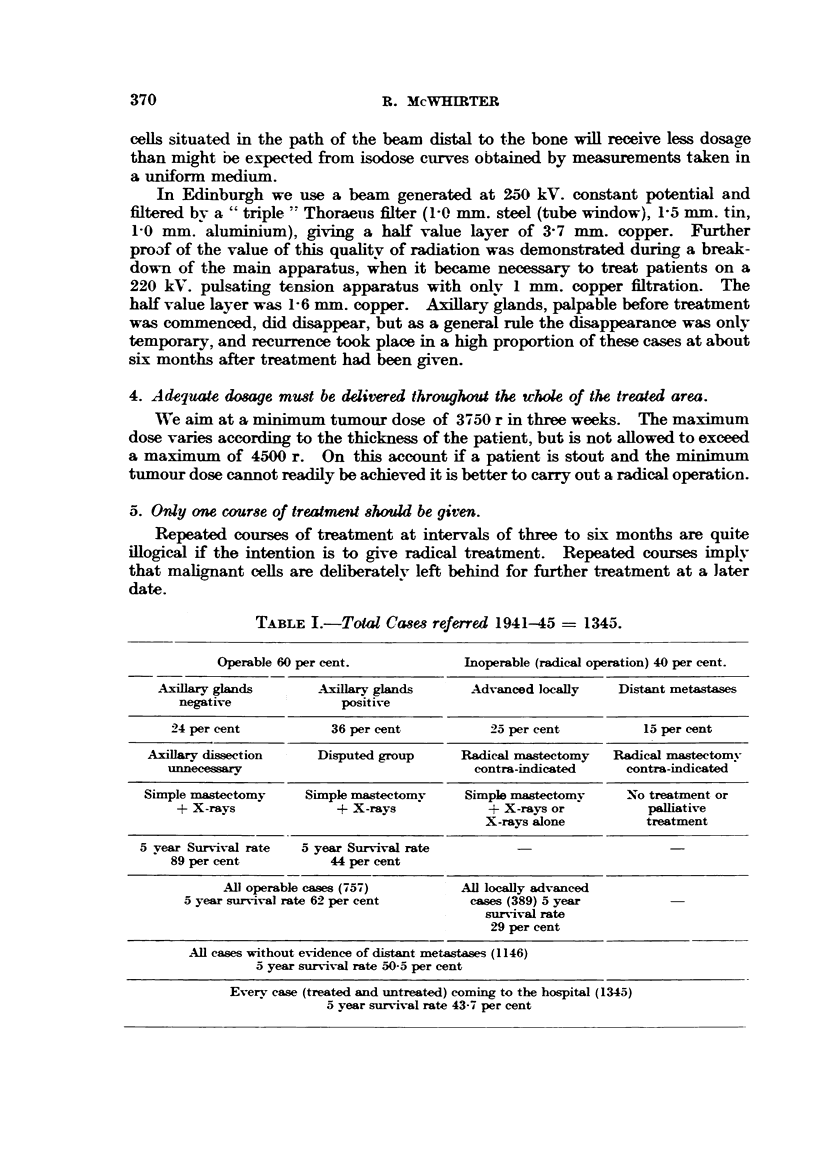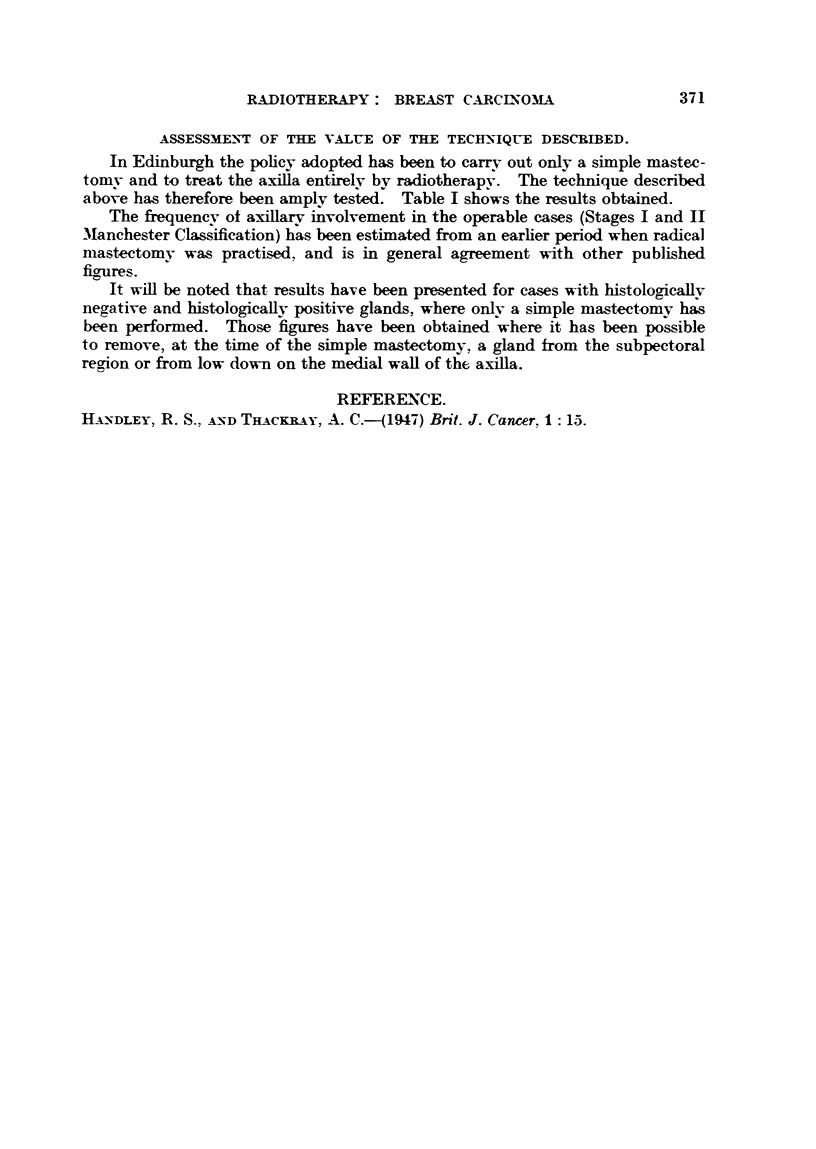# The Principles of Treatment by Radiotherapy in Breast Carcinoma

**DOI:** 10.1038/bjc.1950.35

**Published:** 1950-12

**Authors:** R. McWhirter

## Abstract

**Images:**


### THE PRINCIPLES OF TREATMENT BY RADIOTHERAPY IN BREAST CARCINOMA.

R. McWHIRTER.

*From the Department of Radiotherapy, Royal Infirmary, Edinburgh.*

Received for publication November 23, 1950.

IN only a minority of cases is the disease still confined to the breast by the time the patient is referred to a large general hospital. The surgical results are good so long as the disease is confined to the breast, but immediately the disease extends beyond the breast the results become much poorer. Axillary spread is, of course, common and, in the past, surgical efforts have been concentrated on this route of spread. That this is not the only route of spread, however, has been clearly demonstrated by Handley and Thackray (1947), who have shown that in a high proportion of cases where there is axillary involvement there is also involvement of the glands along the internal mammary artery. Treatment of this route of spread by surgery is not at present possible. In addition the supraclavicular glands are involved in 33 per cent of the cases where the axillary glands are involved and, while surgical removal of the supraclavicular glands may be attempted, the value of this procedure is extremely doubtful. For the majority of cases of breast carcinoma it therefore follows that surgery is unlikely to be successful unless supplemented by radiotherapy.

Success in radiotherapy is dependent on the sensitivity of the tumour to radiation. Unfortunately breast carcinoma is only moderately sensitive, and improved results can only be expected if the radiotherapy is carefully planned.

It is not the purpose of this paper to give a detailed account of the treatment of a patient by radiotherapy, but rather to emphasize the main principles in the treatment of breast carcinoma by radiotherapy. There are five main principles :

1. *The axillary and supraclavicular glands must be treated as one continuous chain.*

In the past it has been customary to regard the supraclavicular glands as being somewhat separate and distinct from the axillary glands. I believe the supraclavicular glands are best regarded as representing the proximal group of glands which accompany the axillary vessels.

If a finger is placed in the apex of the axilla at the time of a radical operation, it can be easily demonstrated that the apex in fact lies almost deep to the supraclavicular region.

In tuberculosis the disease may readily spread from the neck glands to the axillary glands, as is demonstrated in Fig. 1. This radiograph shows the two main groups of glands in the neck—the carotid chain accompanying the carotid vessels and the posterior cervical chain which lies along the anterior border of the trapezius. It will be noted that both chains are continuous with the glands of the axilla, and that the medial group of supraclavicular glands form the proximal group of the axillary chain.

Further proof of the continuity of the neck and axillary glands is shown in

1

2

3

McWhirter.

carcinoma of the thyroid, for if the glands in the neck are involved it is common to find that there is also axillary involvement.

Continuity with the posterior cervical chain is readily demonstrated in patients suffering from carcinoma of the nasopharynx where the posterior cervical chain is commonly involved. If the lower glands of the posterior cervical chain are involved there is nearly always involvement of the axilla.

In the above examples spread downwards has occurred, but it may be assumed that if downward spread can take place the reverse is also possible. As has already been mentioned when the glands in the axilla are involved in breast carcinoma the supraclavicular glands are involved in 33 per cent of cases, and there can be little doubt that in a proportion of cases the supraclavicular glands have become involved by upward spread from the axilla.

It follows therefore that the axillary and supraclavicular glands must be treated as one continuous chain of glands, and the only satisfactory method of doing so is by the use of two directly opposed fields—one field placed in front and the other placed posteriorly as in Fig. 2.

Irradiation of the axilla by a field applied to the base of the axilla and irradiation of the supraclavicular glands by a separate field is unlikely to meet with success because the intervening glands are not treated. It has been stated by some workers that it is difficult to irradiate the supraclavicular region successfully. I believe that in many cases the reappearance of glands in the supraclavicular region has not been due to any difficulty in treating this area, but is due to reinfection of the supraclavicular glands from involved glands high up in the axilla which received no radiation at all.

2. *The internal mammary glands must be treated in continuity with the chest wall.*

This is best done by glancing fields so as to avoid lung damage. The medial glancing field should be placed beyond the midline so as to include within the irradiated area the glands along the internal mammary artery on the side affected (Fig. 3). By using large fields the dose distribution over the area can be made fairly uniform. The use of bolus further assists in obtaining a homogeneous distribution.

The usual separation between the glancing fields is 16 cm., and in radical treatment it is rarely necessary to exceed this distance. If the skin involvement is more extensive and all the diseased area cannot be satisfactorily included within those limits, only palliative treatment is indicated.

It will have been noted that the total area requiring irradiation has been divided into two sections. Every effort is made to avoid a gap between the two sections, and experience has shown that it is uncommon for recurrence to take place along the line of junction. Ideally, of course, it would be better to irradiate the whole of the area to be treated without any division. Unfortunately this cannot be achieved because the axillary and supraclavicular glands cannot be satisfactorily treated except by means of directly opposed fields. (Carcinomata of the axillary tail of the breast do, however, call for some modification of the usual technique.)

3. *Hard quality radiation is essential.*

With radiation of long wave-length much more energy is absorbed by bone than by soft tissue. Not only may this result in bone necrosis, but any tumour cells situated in the path of the beam distal to the bone will receive less dosage than might be expected from isodose curves obtained by measurements taken in a uniform medium.

In Edinburgh we use a beam generated at 250 kV. constant potential and filtered by a " triple " Thoraeus filter (1·0 mm. steel (tube window), 1·5 mm. tin, 1·0 mm. aluminium), giving a half value layer of 3·7 mm. copper. Further proof of the value of this quality of radiation was demonstrated during a breakdown of the main apparatus, when it became necessary to treat patients on a 220 kV. pulsating tension apparatus with only 1 mm. copper filtration. The half value layer was 1·6 mm. copper. Axillary glands, palpable before treatment was commenced, did disappear, but as a general rule the disappearance was only temporary, and recurrence took place in a high proportion of these cases at about six months after treatment had been given.

4. *Adequate dosage must be delivered throughout the whole of the treated area.*

We aim at a minimum tumour dose of 3750 r in three weeks. The maximum dose varies according to the thickness of the patient, but is not allowed to exceed a maximum of 4500 r. On this account if a patient is stout and the minimum tumour dose cannot readily be achieved it is better to carry out a radical operation.

5. *Only one course of treatment should be given.*

Repeated courses of treatment at intervals of three to six months are quite illogical if the intention is to give radical treatment. Repeated courses imply that malignant cells are deliberately left behind for further treatment at a later date.

TABLE I.—*Total Cases referred* 1941–45 = 1345.

| Operable 60 per cent. | | Inoperable (radical operation) 40 per cent. | |
|---|---|---|---|
| Axillary glands negative | Axillary glands positive | Advanced locally | Distant metastases |
| 24 per cent | 36 per cent | 25 per cent | 15 per cent |
| Axillary dissection unnecessary | Disputed group | Radical mastectomy contra-indicated | Radical mastectomy contra-indicated |
| Simple mastectomy + X-rays | Simple mastectomy + X-rays | Simple mastectomy + X-rays or X-rays alone | No treatment or palliative treatment |
| 5 year Survival rate 89 per cent | 5 year Survival rate 44 per cent | — | — |
| All operable cases (757) 5 year survival rate 62 per cent | | All locally advanced cases (389) 5 year survival rate 29 per cent | — |
| All cases without evidence of distant metastases (1146) 5 year survival rate 50·5 per cent | | | |
| Every case (treated and untreated) coming to the hospital (1345) 5 year survival rate 43·7 per cent | | | |

### ASSESSMENT OF THE VALUE OF THE TECHNIQUE DESCRIBED.

In Edinburgh the policy adopted has been to carry out only a simple mastectomy and to treat the axilla entirely by radiotherapy. The technique described above has therefore been amply tested. Table I shows the results obtained.

The frequency of axillary involvement in the operable cases (Stages I and II Manchester Classification) has been estimated from an earlier period when radical mastectomy was practised, and is in general agreement with other published figures.

It will be noted that results have been presented for cases with histologically negative and histologically positive glands, where only a simple mastectomy has been performed. Those figures have been obtained where it has been possible to remove, at the time of the simple mastectomy, a gland from the subpectoral region or from low down on the medial wall of the axilla.

### REFERENCE.

HANDLEY, R. S., AND THACKRAY, A. C.—(1947) *Brit. J. Cancer*, 1 : 15.